# The Science of Homœopathy

**Published:** 1895-08

**Authors:** 


### The Science of Homœopathy; or, A Critical and Synthetical Exposition of the Doctrines of the Homœopathic School. By Charles J. Hempel, M. D. Third edition. Philadelphia: Bœricke & Tafel, 1894.

This well-known book has been before the profession for about twenty years, the present work being its third edition. It is a critical exposition and defense of Homœopathy by a learned man, who is one of the most familiar figures in the new school of medicine.

The author declares that the book is specially written to enlighten the allopathic profession. In his preface he says: "The orthodoxy of an allopathic physician would be doubted by his co-members of medical societies if he dared to publicly acknowledge his belief in the curative efficiency of reasonably small doses of medicine, when administered in accordance with the homœopathic law, *Similia Similibus Curantur;* there is not one of them who has anything but derision and contumely with which to refute the claims of Homœopathy as a therapeutic system of practical value and importance. To these gentlemen of the old school I here offer a volume which will enable them to acquire the knowledge which they are so much in need of, and which will establish some foundation for their claim of being considered 'physicians.' A practitioner armed with this knowledge would cease to be a blind empiric, and would combat the enemy, *Disease*, with all the resources which an enlightened and truly rational science would suggest."

Dr. Hempel does not approve of the high potencies. He enters his solemn protest against them on page xxvii, and calls them a "lamentable and disgraceful perversion of Hahnemann's original method of preparing his attenuations."

All throughout his life he was a bitter and persistent opponent of the high potencies. But he was also an advocate of illogical and glaring perversions of Homœopathy in the direction of rational medicine, and this, consequently, weakened, if it did not completely destroy, the force of his blows at the high potency "perversions." There is, of course, a certain amount of error in the teaching and practicing of high potencies which ought to be eliminated. In this error may be included the revived theory of Isopathy lately introduced into the "regular" school of medicine, and the recently adopted idea of certain practitioners that if a remedy be only potentized, then it becomes a simillimum, whether its symptoms correspond or not with the case in hand. Both of these ideas Dr. Hempel could have materially checked if only his own record had been clear of the charges of "mongrelism" or eclecticism. Unfortunately, his own armor was vulnerable in the part just indicated, and so he was not able to render the efficient service so necessary. Another trouble with Dr. Hempel which weakened him as an authority was his strong partiality for Aconite, which became with him almost a monomania. The writer of this review is well acquainted with physicians of unimpeachable testimony who declare that when they were students sitting under the teaching of Dr. Hempel, he would expend two-thirds of a session in lecturing upon Aconite, and at his clinics would administer Aconite to more than half the patients who came before him. At the examination of a patient he would turn to the class and say, "Gentlemen, what *can* we give him but Aconite?" This, of course, was a glaring folly which seems unexplainable in view of his great learning, sufficiently shown in the book before us.

Dr. Hempel indelibly impressed his name upon the homœopathic school by his extraordinary industry in translating into the English language every one of Hahnemann's writings, and almost every other book upon Homœopathy that was written in the German language. His translations are usually regarded as exceedingly faithful to the originals, and as showing a high order of ability. Notwithstandlng this general opinion, there were not wanting enthusiastic followers of Hahnemann who accused him of inaccuracy in his translations. Perhaps the boldest of these accusers and most implacable antagonist of Dr. Hempel was the late Dr. Lippe. When the writer of this article was a student he had the good fortune to be introduced to the study of materia medica by the lectures of Dr. Lippe, as has been before stated in the editorials of this journal. We well remember the open accusation made by Dr. Lippe that Dr. Hempel did not "approve" of much that Hahnemann taught, and that when, in the course of his translations into English, he met with statements he did not like, he would alter the meaning of the original words so that the translation should give a meaning in accordance with his own ideas. Then the lecturer would exclaim, with "dramatic emphasis," that no earnest seeker after the truth of Homœopathy should ever read any book "translated by Charles Julius Hempel," whom he further denominated "a penny-a-liner," and then, as if addressing a student whom he had caught with one of the forbidden books, he would say, "You must not read that book," and picking up his book of lecture notes, would put it under his arm and make a motion as if walking off with it to illustrate his contempt of Dr. Hempel's writings. Whether these accusations of Dr. Lippe were really true, especially to the extent that Dr. Lippe claimed, we are unable to say, but the above reminiscence is brought to mind by reading Dr. Hempel's book, and it is given to the profession as an interesting item that throws some light upon the characteristics of both Hempel and Lippe.

Notwithstanding, Dr. Hempel has rendered a great service to Homœopathy, and it is not easy to get hold of any of the older homœopathic works that he has not translated. They are standard works to-day, and are likely to continue so until a more able and thorough materia medica, clearing up all doubtful points, shall be published, and thus displace them.

